# LncRNA PVT1 up-regulation is a poor prognosticator and serves as a therapeutic target in esophageal adenocarcinoma

**DOI:** 10.1186/s12943-019-1064-5

**Published:** 2019-10-10

**Authors:** Yan Xu, Yuan Li, Jiankang Jin, Guangchun Han, Chengcao Sun, Melissa Pool Pizzi, Longfei Huo, Ailing Scott, Ying Wang, Lang Ma, Jeffrey H. Lee, Manoop S. Bhutani, Brian Weston, Christopher Vellano, Liuqing Yang, Chunru Lin, Youngsoo Kim, A. Robert MacLeod, Linghua Wang, Zhenning Wang, Shumei Song, Jaffer A. Ajani

**Affiliations:** 1 Departments of Gastrointestinal Medical Oncology, The University of Texas MD Anderson Cancer Center, 1515 Holcombe Blvd., Houston, TX 77030 USA; 2 Department of Surgical Oncology and General Surgery, First Hospital of China Medical University, Shenyang, 110001 People’s Republic of China; 3 Departments of Genomic Medicine, The University of Texas MD Anderson Cancer Center, Houston, TX 77030 USA; 4 Departments of Molecular & Cellular Oncology, The University of Texas MD Anderson Cancer Center, Houston, TX 77030 USA; 5 Departments of Gastroenterology&Hepatology, The University of Texas MD Anderson Cancer Center, Houston, TX 77030 USA; 6 Center for Co-Clinical Trial, The University of Texas MD Anderson Cancer Center, Houston, TX 77030 USA; 7 Ionis Pharmaceuticals, Inc. 2855 Gazelle Court, Carlsbad, CA 92010 USA

**Keywords:** Lnc RNA, PVT1, YAP1, Antisense oligonucleotides, Esophageal adenocarcinoma

## Abstract

**Background:**

PVT1 has emerged as an oncogene in many tumor types. However, its role in Barrett’s esophagus (BE) and esophageal adenocarcinoma (EAC) is unknown. The aim of this study was to assess the role of PVT1 in BE/EAC progression and uncover its therapeutic value against EAC.

**Methods:**

PVT1 expression was assessed by qPCR in normal, BE, and EAC tissues and statistical analysis was performed to determine the association of PVT1 expression and EAC (stage, metastases, and survival). PVT1 antisense oligonucleotides (ASOs) were tested for their antitumor activity in vitro and in vivo.

**Results:**

PVT1 expression was up-regulated in EACs compared with paired BEs, and normal esophageal tissues. High expression of PVT1 was associated with poor differentiation, lymph node metastases, and shorter survival. Effective knockdown of PVT1 in EAC cells using PVT1 ASOs resulted in decreased cell proliferation, invasion, colony formation, tumor sphere formation, and reduced proportion of ALDH1A1^+^ cells. Mechanistically, we discovered mutual regulation of PVT1 and YAP1 in EAC cells. Inhibition of PVT1 by PVT1 ASOs suppressed YAP1 expression through increased phosphor-LATS1and phosphor-YAP1 while knockout of YAP1 in EAC cells significantly suppressed PVT1 levels indicating a positive regulation of PVT1 by YAP1. Most importantly, we found that targeting both PVT1 and YAP1 using their specific ASOs led to better antitumor activity in vitro and in vivo.

**Conclusions:**

Our results provide strong evidence that PVT1 confers an aggressive phenotype to EAC and is a poor prognosticator. Combined targeting of PVT1 and YAP1 provided the highest therapeutic index and represents a novel therapeutic strategy.

**Electronic supplementary material:**

The online version of this article (10.1186/s12943-019-1064-5) contains supplementary material, which is available to authorized users.

## Introduction

Esophageal cancer (ESCA) is one of the most aggressive malignancies and ranks seventh in terms of incidence and sixth in mortality, globally [1]. Despite recent clinical advances, patients continue to have a poor prognosis. Esophageal adenocarcinoma (EAC), a major subtype of ESCA, has increased dramatically in incidence in recent decades [2, 3]. In Western countries, the incidence of EAC has exceeded that of the previously more common esophageal squamous cell carcinoma (ESCC) [2, 3]. Besides different etiologic and epidemiologic factors, EAC and ESCC are distinct in their molecular characteristics. EAC is characterized by frequent somatic DNA structural rearrangements (copy number variations, CNVs) similar to the chromosomal instability (CIN) subtype of gastric adenocarcinoma [4]. Amplification of 8q24 is one of the most frequent events in many cancers including EAC. In recent years, increasing evidence suggests that the plasmacytoma variant translocation 1 (PVT1) gene, which maps to 8q24, plays an oncogenic role through either amplification or overexpression. PVT1 is the first long non-coding RNA (lncRNA) identified in human cancer [5, 6]. Previous studies of PVT1 revealed that there are various mechanisms that contribute to carcinogenesis in cancers [7–9]. Zhao and colleagues showed that PVT1 binds to the signal transducer activator phospho-STAT3 protein and protects it from poly-ubiquitin-proteasomal degradation to enhance the activation of STAT3 signaling pathway, thereby increasing VEGFA expression to induce angiogenesis. In turn, the activated STAT3 triggers PVT1 transcription to sustain the oncogenic effect of the PVT1/STAT3/VEGFA axis continuously [7]. Additionally, PVT1 has been found to promote gastric cancer progression through a positive feedback regulation with FOXM1 [10]. However, its role and mechanisms in EAC progression remains largely unknown.

Inhibition of LncRNA has been reported as a potential therapeutic strategy in some human diseases including cancer [11]. Antisense oligonucleotide (ASO) is an effective and feasible way to target a gene of interest selectively and four ASOs (fomivirsen, nusinersen, mipomersen, and eteplirsen) have already been approved by the FDA [12–15]. In contrast to double-stranded siRNAs, ASOs are single stranded and amphipathic, which facilitates cell uptake without the need for transfection, and they distribute broadly after systemic administration [16]. Moreover, ASOs have proven to cleave target RNA in both the cytoplasm and nucleus depending on RNase H1 [17]. However, whether targeting PVT1 using the PVT1 ASOs is efficient in EAC has not been reported.

In this study, we investigated the status of PVT1 amplifications and/or expression in EAC progression from normal esophageal tissue to Barrett’s epithelium (BE) to EACs and the association with malignant phenotype; and also reviewed The Cancer Genome Atlas (TCGA) database. The anticancer effect of silencing PVT1 with ASOs was examined in vitro and in vivo. In addition, we discovered a positive regulation between PVT1 and YAP1 in EAC, not previously described. Simultaneously blocking PVT1 and YAP1 resulted in the most effective antitumor strategy. Our data demonstrate that PVT1 plays a critical role in EAC progression and that PVT1 is a potential therapeutic target.

## Materials and methods

### Patient specimens and cell lines

Specimens from 156 patients were obtained during an endoscopic exam at the University of Texas M. D. Anderson Cancer Center (MDACC, Houston, TX) under an Institutional Review Board approved protocol. All specimens were snap-frozen and stored at − 80 °C until use. Clinical characteristics of patients were collected from an established database. Normal esophageal epithelial cell line HET-1A was purchased from ATCC. Four esophageal adenocarcinoma cancer cell lines (JHESO, OE19, FLO1, and SKGT-4) were kindly provided by Drs. Mien-Chie Hung and Health Skinner (MDACC) and described previously [18, 19]. All human cell lines were authenticated at the Characterized Cell Line Core Facility of MDACC every 6 months. Doxycycline-inducible YAP1 lentiviral plasmid (PIN20YAP1) was constructed by inserting flag-tagged YAP1^S127A^ cDNA amplified from CMV-S127A-YAP into pINDUCER20 (provided by Thomas Westbrook, Baylor College of Medicine, Houston, TX). Antisense oligonucleotides (ASOs) with next generation chemistry (constrained ethyl = cEt) were synthesized as described previously [20]. The lentiCRISPR target YAP1 was constructed using lentiCRISPR v2 (Addgene plasmid No. 52961). Guide RNAs design follows Dr. Feng Zhang’s website (http://crispr.mit.edu/; Massachusetts Institute of Technology). All cells were cultured in the DMEM medium (Sigma-Aldrich, St Louis, MO) supplemented with 10% fetal bovine serum, and maintained in a humidified incubator at 37 °C with 5% CO_2_.

### Flow cytometry

Expression of ALDH1A1 was determined by flow cytometry using ALDEFLUOR™ kit (Stem Cell Technologies, Vancouver, Canada), as per manufacturer’s instructions. Briefly, cultured human EAC cells were suspended in ALDEFLUOR™ assay buffer, and 1 × 10^6^ cells/ml cell suspension was added to a tube containing 5 μL of the ALDH1A1 substrate. As a negative control, a 0.5 ml aliquot from each sample was treated with an ALDH1A1-specific inhibitor. Following 30 min incubation at 37 °C and centrifugation, the cells were washed with the assay buffer, followed by resuspension in 0.3 ml of ice cold ALDEFLUOR™ assay buffer for flow labeling (FACSCalibur™; BD Biosciences, San Jose, CA).

### Protein extraction and western blot analysis

Proteins were isolated from EAC cell lines as indicated and analyzed using Western blotting as described previously [21].

### RNA extraction and qPCR

Total RNA was extracted using Trizol (Ambion, Austin, TX) according to manufacturer’s instructions. After reverse transcription using SuperScript IV First-Strand Synthesis System (Invitrogen, Carlsbad, CA), quantitative real-time PCR (qPCR) was performed using SYBR® Select Master Mix (Applied Biosystems®) on the Applied Biosystems 7500 Fast platform (Applied Biosystems, CA). PVT1 and other related molecules were detected using the primers as described in Additional file 5: Table S1.

### Confocal immunofluorescence

The immunofluorescence staining was performed on OE19 and JHESO EAC cells treated with PVT1 ASO at 1 μM as performed as previously described [18]. Expression and localization of the proteins were observed under a confocal microscope system (FluoView FV500; Olympus) and analyzed by CellQuest PRO software (BD Biosciences, San Jose, CA). Information for antibodies used in immunofluorescent staining and western blot was described in Additional file 6: Figure S6.

### Colony formation

OE19 and JHESO EAC cells were plated in the 6-well plates and exposed to PVT1 ASOs and/or YAP1 ASOs without any transfection agent (free uptake) at the dosage indicated and cultured for 10 to 14 days to allow for colony formation. Cells were then fixed in a 10% formalin solution. Images were taken after 3% crystal violet staining. Colony numbers and average colony sizes were determined using Image J [22].

### Cell migration assay

The invasive capacity of cells was determined by using the Matrigel-coated invasion chambers with an 0.8-mm pore size (BD Biosciences, San Jose, CA). A single-cell suspension from JHESO or OE19 EAC cells containing 1 × 10^5^ cells was added to the inner chamber. PVT1 ASOs were added to the lower channel of medium as indicated dosage. After incubation for 24 h at 37 °C in 5% CO_2_, cells on the upper surface of the inner chamber were removed with cotton swabs. Cells that adhered to the lower surface of the membrane, representing invaded cells, were fixed, stained with Diff-Quik (Dade Behring Inc., Deerfield, IL) and counted.

### Tumor sphere formation assay

JHESO or OE19 EAC cells (2500/well) with or without exposure to PVT1 ASOs were seeded in triplicate onto a 24-well ultra-low attachment plate (Corning) in serum-free DMEM/F-12 supplemented with 10 ng/ml epidermal growth factor, 5 mg/mL insulin, 0.5 mg/ml hydrocortisonum, and bovine pituitary extract (Invitrogen, Carlsbad, CA). After 10 to 14 days of culture, the number of tumor spheres formed (diameter > 100 μm) was counted under a microscope.

### Reverse-phase protein arrays (RPPA)

The RPPA analysis was performed in lysates of three EAC cell lines-JHESO, OE19 and Flo-1 control cells and PVT1 ASO treatment for 72 h by the MDACC Functional Proteomics Core Facility using a total of 298 antibodies. Normalized RPPA data in log2 values were used for comparison protein expression between vehicle control and ASO treatment. Clustering analysis was performed using JMP 14.0 (SAS Institute Inc., Cary, NC).

### RNA immunoprecipitation (RIP) assay

RIP was performed in native conditions as described [23]. Briefly, 1 × 10^7^ JSHEO and SKGT-4 cells were washed with cold PBS twice, and then pelleted and lysed in 1 ml ice-cold polysomal lysis buffer (100 mM KCl, 5 mM MgCl2, 10 mM HEPES pH 7.0, 0.5% NP-40, 1 mM DTT supplemented with the protector RNase inhibitor and protease inhibitor cocktail. Turbo DNase (200 U) was then added to the lysate and incubated on ice with rotation for 30 min. The cell lysate was diluted in the NT2 buffer (50 mM Tris-HCl pH 7.4, 150 mM NaCl, 1 mM MgCl2, 0.05% NP-40) and 50 μl of the supernatant was saved as input for PCR analysis. 50 ul protein G magnetic beads were washed twice by the NT2 buffer, then pre-blocked by 1× PBS + 5 mg ml^− 1^ BSA, and incubated with 5 μg of rabbit IgG and YAP antibodies with rotation at room temperature for 1 h. 500 μl of the supernatant was incubated with antibodies binding beads at 4 °C overnight with rotation. The RNA/antibody complex was washed six times by NT2 buffer supplemented with protector RNase inhibitor and protease inhibitor cocktail. The RNA was extracted using acid-phenol: chloroform, pH 4.5 (125,24:1) according to the manufacturer’s protocol and subjected to RT-qPCR analysis.

### In vivo xenograft mouse model

In a JHESO xenograft model of EAC, 1 × 10^6^ JHESO cells were subcutaneously injected into nude mice (*n* = 5/group). After about 10 days, the mice underwent subcutaneous injection of control ASO, PVT1 ASO (50 mg/kg), YAP1 ASO (50 mg/kg), or a combination of them (25 mg/kg/mouse, each) three times a week for at least 3 weeks. Tumor sizes were measured with a digital caliper (VWR International) once tumors reached a visible size, and tumor volume was determined by the formula: tumor volume (mm^3^) = [length (mm) × width (mm)^2^] × 0.52 [24].

### Statistical analysis

Data were analyzed using the Student t test and Fisher exact test (for colony formation, cell migration assay). The Kaplan–Meier method was used to estimate probability of survival. The log rank test and Cox model were used to determine the association between markers and survival outcomes. Other assays are presented in graphs as mean ± SEM. and represent the results of at least three experiments. The significance of differences between the groups was judged using a two-tailed Student t-test. Results were considered statistically significant if the *P* value was less than 0.05. All tests were done with GraphPad Prism 7 software (GraphPad Software, Inc.).

## Results

### Overexpression of PVT1 lncRNA in EAC patients associated with poor prognosis

**Fig. 1:**
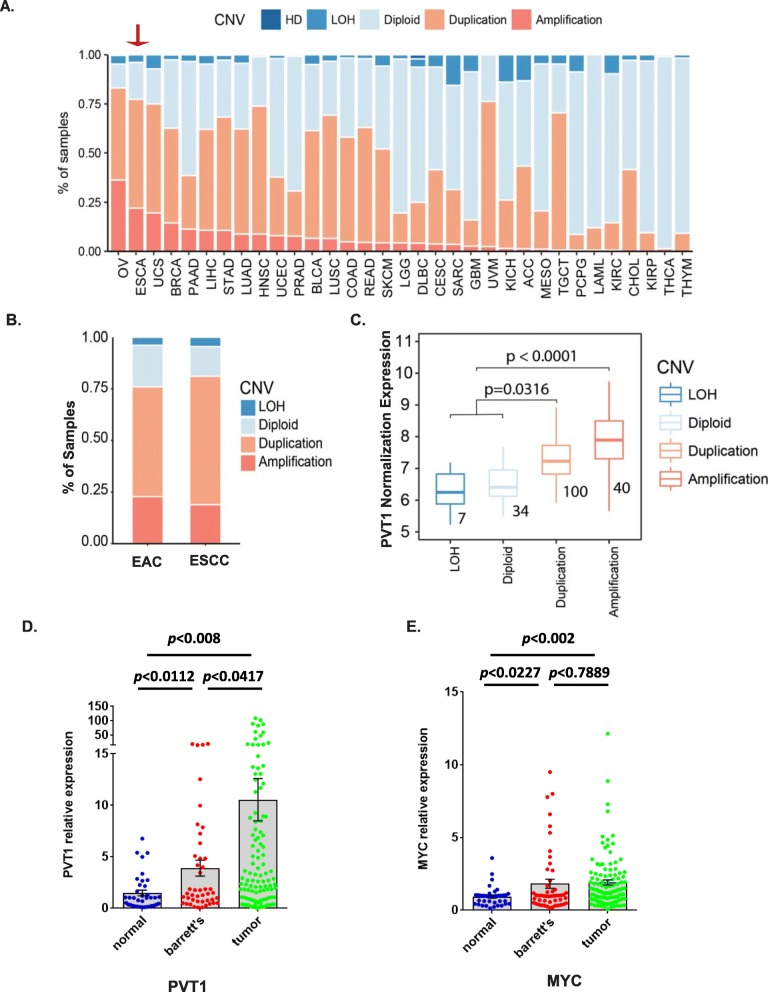
LncRNA PVT1 is amplified and overexpressed in EAC tumor tissues compared to Barrett’s epithelium and normal tissues. **a.** Analysis of PVT1 genomic alteration in TCGA dataset revealed PVT1 was amplified in over 20% of ESCA cases, and around 75% of ESCA cases containing both amplification and duplication (> 3 N) in total. **b.** Amplification of PVT1 gene in EAC is more common than that in ESCC. **c.** PVT1 expression levels significantly higher in amplification and duplication cases than that in LOH and diploid cases. **d** & **e.** Expressions PVT1 lncRNA and MYC mRNA were measured by qPCR and normalized to GAPDH in our own patient-cohort (156 cases)

First, genomic alteration of PVT1 was analyzed using the TCGA dataset across multiple cancer types; we found that ESCA was the second-ranking cancer type with high PVT1 alterations with 20% PVT1 amplification and around 75% of ESCA cases contained both amplification and duplications (> 3 N) (Fig. 1a). On further analysis of two major ESCA subtypes EAC and ESCC, we found that EAC patients had relative higher PVT1 amplification and its higher incidence in Western population leading us to focus on EAC (Fig. 1b). When integrated with TCGA RNA Seq data in ESCA, genetic alterations of PVT1 either duplication or amplification were significantly associated with PVT1 expression level (Fig. 1c), indicating that genetic alterations of PVT1 in EC lead to its up-regulation in mRNA level.

**Fig. 2:**
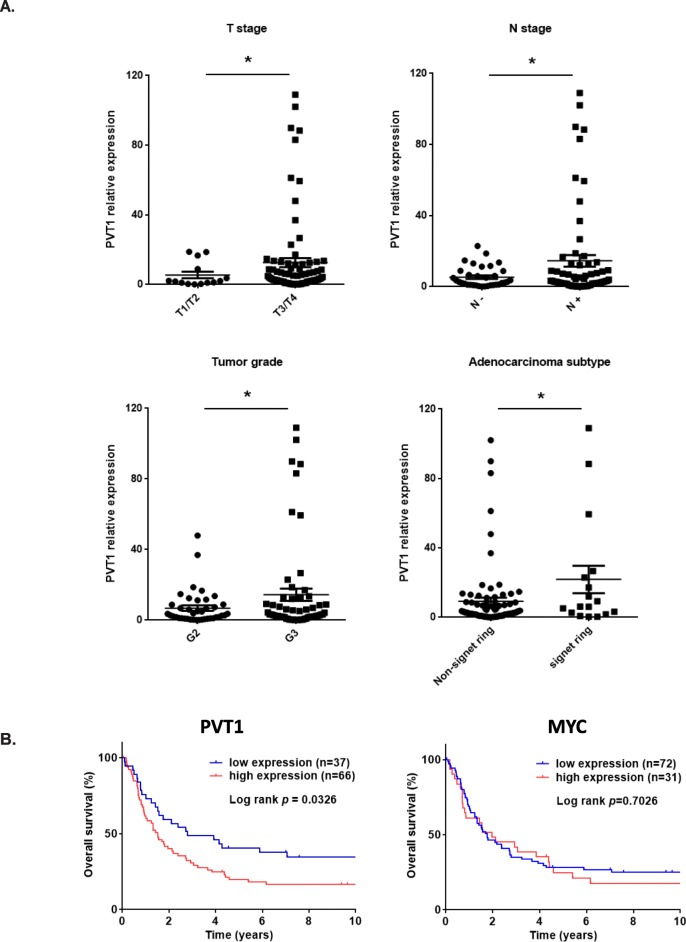
Correlation between expression of PVT1 and clinical characteristics. **a.** Higher expression of PVT1 lncRNA was significantly correlated with advanced tumor stage (T stage and N stage), as well as higher grade tumor and signet ring cell subtype. **b.** Kaplan-Meier analysis of OS time according to PVT1 and MYC expression level. EAC patients with higher PVT1 expression showed poorer survival than that of low expression (left). MYC expression is not associated with survival of patients with EAC. High expression was defined as ≥2 times of mean of normal tissue, whereas low expression was considered as < 2 times of mean of normal tissue. Log-rank probabilities between high and low expression were shown. Error bars, mean ± SEM. *, *P* < 0.05

To confirm the discovery from the TCGA dataset, next we measured the expression of PVT1 lncRNA in 37 normal tissues, 56 BEs, and 103 EACs from 156 patients (113 with EAC, 42 with other diagnosis) by qPCR in our own patient cohort. The result showed that increased expression of PVT1 lncRNA was significantly correlated with progression of BE (BE vs. normal tissues, *p* = 0.0112; EAC vs. normal tissues, *p* = 0.008; and EAC vs. BE, *p* = 0.0417) (Fig. 1d). Interestingly, we found that PVT1 expression was significantly associated with clinical characteristics. As shown in Fig. 2a, higher PVT1 expression was significantly associated with higher T stage (*p* = 0.0369), higher N stage (*p* = 0.0484), and unfavorable tumor grade (*p* = 0.0428). Higher expression of PVT1 was found frequently in EACs with signet ring cells, which are more aggressive subtypes than those without signet ring cells (*p* = 0.0315). Furthermore, when EAC patients (*n* = 113) were stratified according to the PVT1 lncRNA expression levels using the average expression of normal tissues as the cut-off point: in the high expression (*n* = 66, fold changes ≥2) and low expression groups (*n* = 37, fold changes < 2), the Kaplan-Meier analysis revealed that EAC patients with higher PVT1 lncRNA expression had shorter overall survival (OS) than those who had the lower PVT1 lncRNA expression (Fig. 2b).

MYC is an important gene involved in many important processes of tumor progression and is located next to the PVT1 locus. In comparison, we determined the MYC levels using qPCR in our same patient cohort as done with PVT1. Although the MYC mRNA levels were increased in BE’s progression, which was significantly higher in BE compared with normal tissues (*p* = 0.0227), and were much higher in EACs compared with normal tissues, (Fig. 1e) but there was no association between tumor grade, N stage, and survival and MYC expression (Additional file 1: Figure S1 and Fig. 2b, right panel). These results indicate that PVT1 is more critical and relevant to BE’s progression than MYC is.

### Inhibition of PVT1 suppresses EAC cell growth in vitro and in vivo

**Fig. 3:**
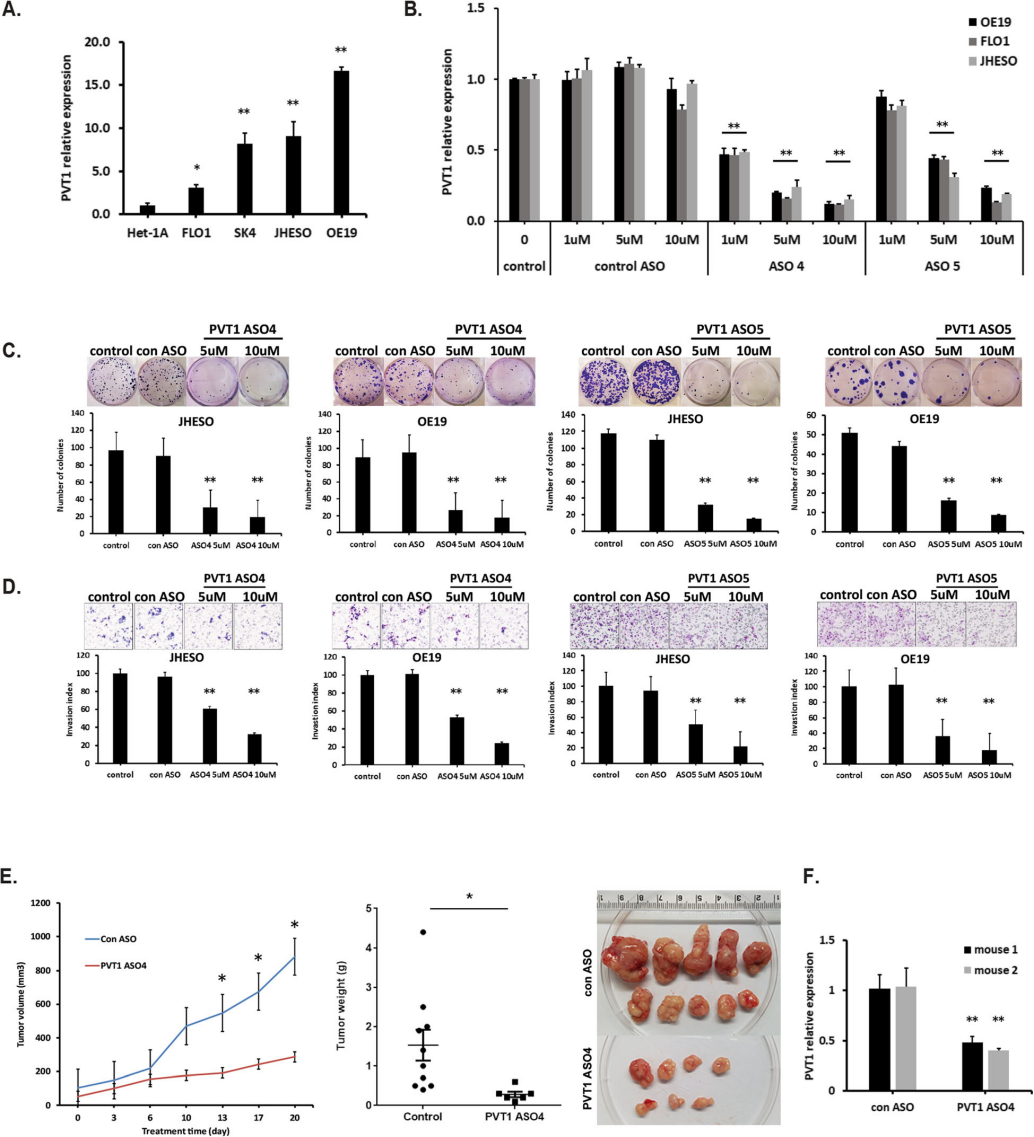
PVT1 suppression by PVT1 ASOs inhibits EAC cells growth in vitro and in vivo. **a.** PVT1 expression in EAC cell lines (FLO1, SK-GT-4, JHESO, and OE19) and normal esophageal epithelial cell line HET-1A were determined by realtime qPCR analysis. **b.** PVT1 expression was analyzed by qPCR in PVT1 ASOs treated EAC cell lines. PVT1 specific antisense oligonucleotides (ASO4 and ASO5) reduced PVT1 expression in dose-dependent manner in three EAC cell lines. **c** and **d.** Inhibition of PVT1 by ASOs significantly suppressed colony formation (**c**) and decreased cell invasion (**d**) in both JHESO and OE19 cells in two individual PVT1 ASOs respectively. **e**. Average tumor volume in mice that treated with PVT1 ASO4 or control ASO via subcutaneous injection for 3 weeks (left). Actual tumor weights were retrieved from the mice at the termination of the experiment (middle). Representative tumors after scarified were shown (right). **f.** The expression of PVT1 in mouse tumor tissues measured by qPCR has been shown significant reduction upon treatment with PVT1 ASO4 compared to the control group. Error bars, mean ± SEM. *, *P* < 0.05; **, *P* < 0.01

PVT1 expression was first detected in an immortalized normal esophageal epithelial cell line (HET-1A) and four EAC cell lines (FLO1, SK-GT-4, JHESO, and OE19) by qPCR. The level of PVT1 lncRNA in EAC cell lines was markedly increased compared with the HET-1A cell line (Fig. 3a). Subsequently, we evaluated human PVT1-specific ASOs and found three PVT1 ASOs (ASO 4, ASO 5 and ASO 6) that efficiently inhibited PVT1 expression in a dose-dependent manner in the EAC cell lines (Fig. 3b & Additional file 2: Figure S2A).

To further elucidate the functional effect of PVT1 inhibition by PVT1 ASOs in EAC cells, colony formation and cell migration assay were conducted in JHESO and OE19, two EAC cells with constitutively high PVT1 levels exposed to two PVT1 ASOs (ASO4 and ASO5, respectively) at the dosage indicated. The results showed that both PVT1 ASOs could significantly suppress colony formation in a dose-dependent manner in both EAC cells compared with untreated or control ASO-treated cells (Fig. 3c). Similarly, cell invasion assay showed that the number of invading cells was significantly decreased when EAC cells were treated with PVT1 ASOs (Fig. 3d).

To further investigate whether PVT1 inhibition by PVT1 ASO could impact tumor growth in vivo, nude mice were subcutaneously injected with JHESO cells. Mice were randomly assigned to two groups: those treated with control ASO and those treated with PVT1 ASO (50 mg/kg, 3 times/week, subcutaneously injected into the back of neck), respectively. Tumor growth and tumor volumes were observed and measured over 3 weeks of treatment. The volumes and weights of tumors from PVT1 ASO treated group exhibited a significant reduction compared with that of the control group (Fig. 3e). In addition, the expression of PVT1 as measured by qPCR in tumor tissues showed significant reduction when treated with PVT1 ASO compared with the control ASO group (Fig. 3f). These results confirmed that inhibition of PVT1 by PVT1 ASO efficiently suppressed EAC growth in vitro and in vivo*.*

### PVT1 inhibition by PVT1 ASOs reduces CSC-characteristics of EAC cells

**Fig. 4:**
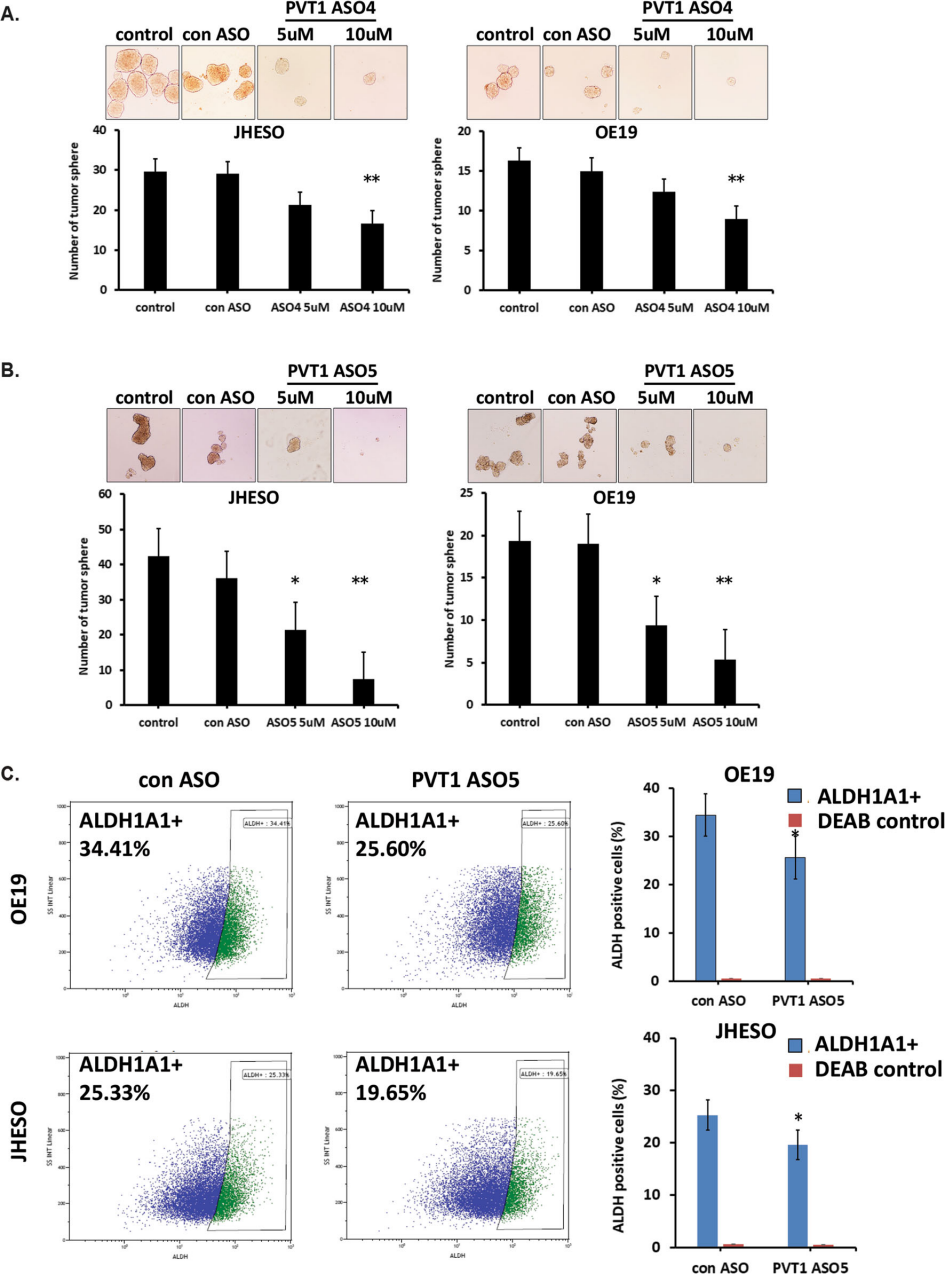
Suppression of PVT1 by ASOs reduces cancer stem cell (CSC) properties in EAC cells. **a** and **b**. Representative images (top) and the number (below) of tumorsphere formation showed that silencing of PVT1 by ASO4 and ASO5 suppressed sphere formation in both JHESO and OE19 cells. **c**. PVT1 ASO5 reduced the population of ALDH1+ cells (top: OE19; below: JHESO). JHESO and OE19 cells treated with PVT1 ASO5 at 10uM for 48 h and then labeling with ALDH1A1 using ALDH1 Labeling Kit. DEAB as negative control. Scale bar, 100 μm. Error bars, mean ± SEM. *, *P* < 0.05; **, *P* < 0.01

To assess the role of PVT1 on CSC-properties of EAC cells, a tumor sphere assay was performed on JHESO and OE19 cell lines exposed to PVT1 ASOs. Figures 4a & b show that tumor sphere formation in both JHESO and OE19 cells were significantly reduced upon treatment with PVT1 ASOs compared with that in EAC cells treated with control ASO.

There is increasing evidence that intracellular marker ALDH1A1 is associated with the CSC phenotype in different types of cancer [25, 26]. In this study, the population of ALDH1+ EAC cells treated with or without PVT1 ASO was determined by flow cytometry using ALDEFLUOR™ kit. We found the proportion of ALDH1+ population in EAC cell lines (JHESO and OE19) was around 30%, which is consistent with a prior report [25]. While PVT1 knockdown by ASOs greatly decreased the population of ALDH1A1+ cells (Fig. 4c). In addition, reduced expression of ALDH1 was in concert with a significant reduction in tumor sphere size and their numbers. These results suggested that PVT1 lncRNA is associated with CSC-related properties of EAC and may play a critical role in EAC tumorigenesis/progression.

### Positive feedback regulation of PVT1 by YAP1 in EAC

**Fig. 5:**
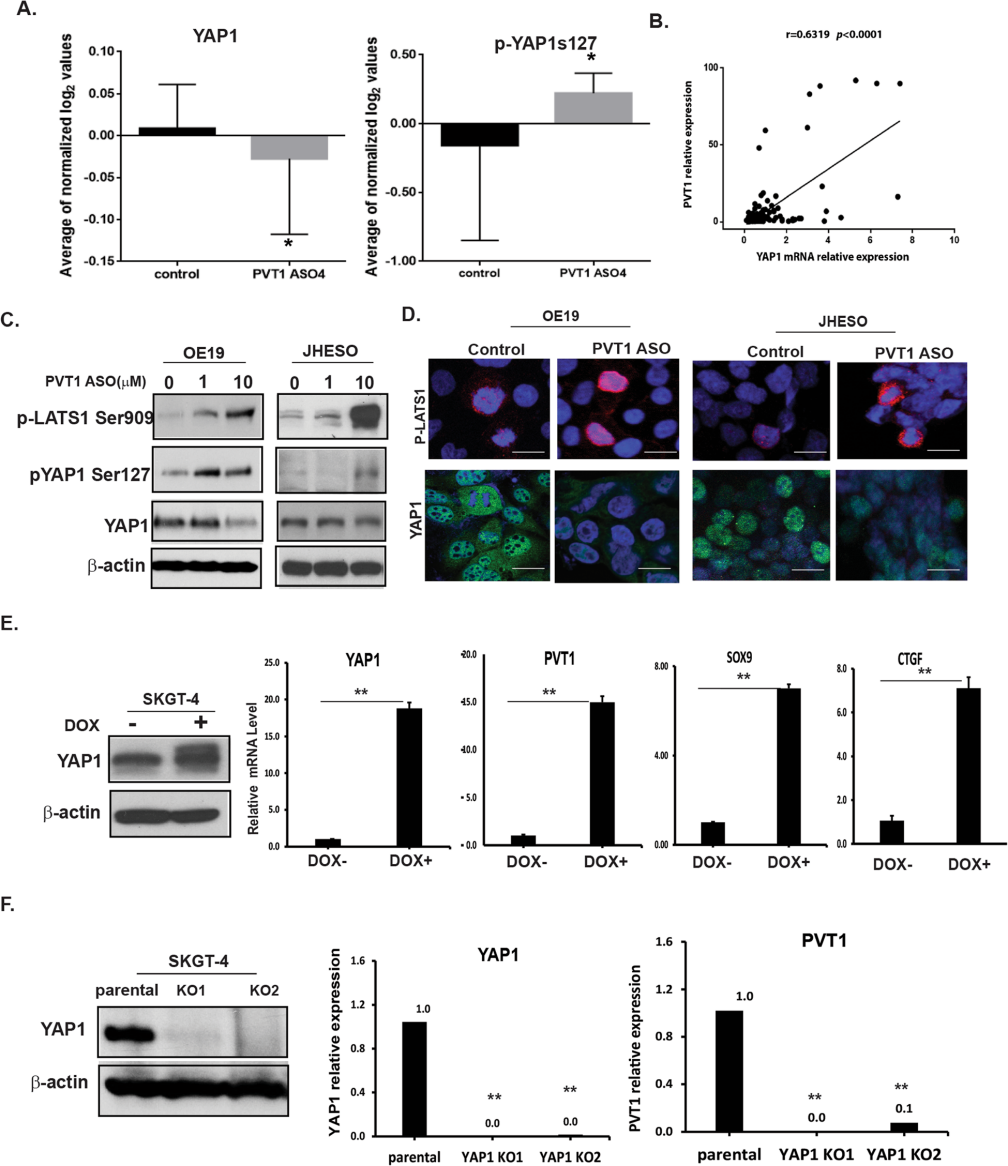
Mutual regulation of PVT1 and YAP1 in EAC cells. **a**. Reverse-phase protein arrays (RPPA) revealed changes in total YAP1 (left) and phosphorylated YAP1 (Ser 127) (right) treated with or without PVT1 ASOs. *p < 0.05. **b**. Correlation analysis (Pearson’s correlation) between PVT1 and YAP1 expression in 103 EAC patients. Pearson’s correlation (scatter plot) of expression levels between PVT1 and YAP1 in patients with EAC (r = 0.6319, *p* < 0.0001). **c**. Total YAP1 level and phosphor-YAP1 (Ser 127) or phosphor-LATS1(Ser 909) were determined by Western blot in OE19 and JHESO cell lines treated with PVT1 ASO at concentration indicated. **d**. Immunofluorescent staining of YAP1 and phosphor-LATS1 in OE19 and JHESO cell lines treated with PVT1 ASO. Scale bar; 25 μm. **e**. Level of YAP1 and its targets-SOX9 and CTGF as well as PVT1 level were determined by Western blot and qPCR in SKGT-4 cells with or without YAP1 induction (Doxycycline; DOX+)., **f**. YAP1 and PVT1 level was detected by q-PCR or western blot in YAP1 knockout clones (YAP1 KO1 and YAP1 KO2) with LentiCRISPR/Cas9 compared to control cells. PVT1 expression was dramatically decreased (bottom) upon knock out YAP1 in SKGT-4 EAC cells. Error bars, mean ± SEM. ** *P* < 0.01

To identify molecular pathways involved in regulation of PVT1 in EAC, an RPPA analysis of multiple signaling pathways was performed on untreated and treated EAC cell lines (JHESO, OE19, and FLO1) with PVT1 ASO. The results showed that the expression of YAP1 has been markedly downregulated in PVT1 ASO-treated cells compared to the control cells (Fig. 5a, left panel); conversely phosphorylated-YAP1 (Ser 127) increased (Fig. 5a, right panel) indicating YAP1 as a potential down-stream target of PVT1. The association between PVT1 and YAP1 expression was further confirmed in our patient-cohort by qPCR that PVT was significantly correlated with YAP1 expression (Fig. 5b). Pearson’s correlation analysis indicated that there is a strong positive correlation between expression of YAP1 and PVT1 (r = 0.6319, *p* < 0.0001) (Fig. 5b). To further confirm the positive correlation between PVT1 and YAP1, PVT1 ASOs were used as a tool to determine the change of YAP1 upon suppression of PVT1 in EAC cell lines OE19 and JHESO. As shown in Fig. 5c, phosphorylated-LATS1 (Ser 909), a direct upstream kinase of YAP1 that controls YAP1 phosphorylation and degradation, was increased in a dose dependent manner upon treatment with PVT1 ASO in two EAC cell lines. Correspondingly, phosphorylated-YAP1 at Ser 127 was markedly increased as well in JHESO and OE19 cells treated with PVT1 ASO, while the expression of YAP1 decreased (Fig. 5c). The immunofluorescent staining further confirmed that nuclear expression of YAP1 was dramatically reduced, while phosphorylation of LATS1 was increased (Fig. 5d). These results were consistent with the canonical Hippo signaling regulatory cascades that LATS1/2 is to be phosphorylated and activated, thereby induce YAP1 phosphorylation and inactivation.

To verify whether YAP1 in turn impacts on the PVT1 expression, we generated inducible YAP1 cDNA expression in SKGT-4 EAC cells. As shown in Fig. 5e, successful induction of YAP1 significantly increased PVT1 expression which correspondingly associated with increased YAP1 levels and also its downstream targets-SOX9 and CTGF (Fig. 5e); In contrast, efficient knockout YAP1 in SKGT-4 cells by CRISPR/Cas9 genome editing (Fig. 5f, left panel) in two clones (YAP1 KO1 and YAP1 KO2) dramatically decreased PVT1 expression which is correlated with decreased YAP1 protein and mRNA level (Fig. 5f). To test the association between YAP and PVT1, we applied an RNA immunoprecipitation (RIP) assay using the YAP antibody, amplifying PVT1 using two individual primers and demonstrated that YAP was associated constitutively with PVT1 in both SKGT-4 and JHESO EAC cells (Additional file 4: Figure S4). Altogether, these data suggested both PVT1 and YAP1 are associated and positively regulate each other, which may provide a novel mechanistic insight for treatment of EAC.

### Simultaneous inhibition of PVT1 and YAP1 leads to enhanced growth inhibition of EAC cells in vitro and in vivo

**Fig. 6:**
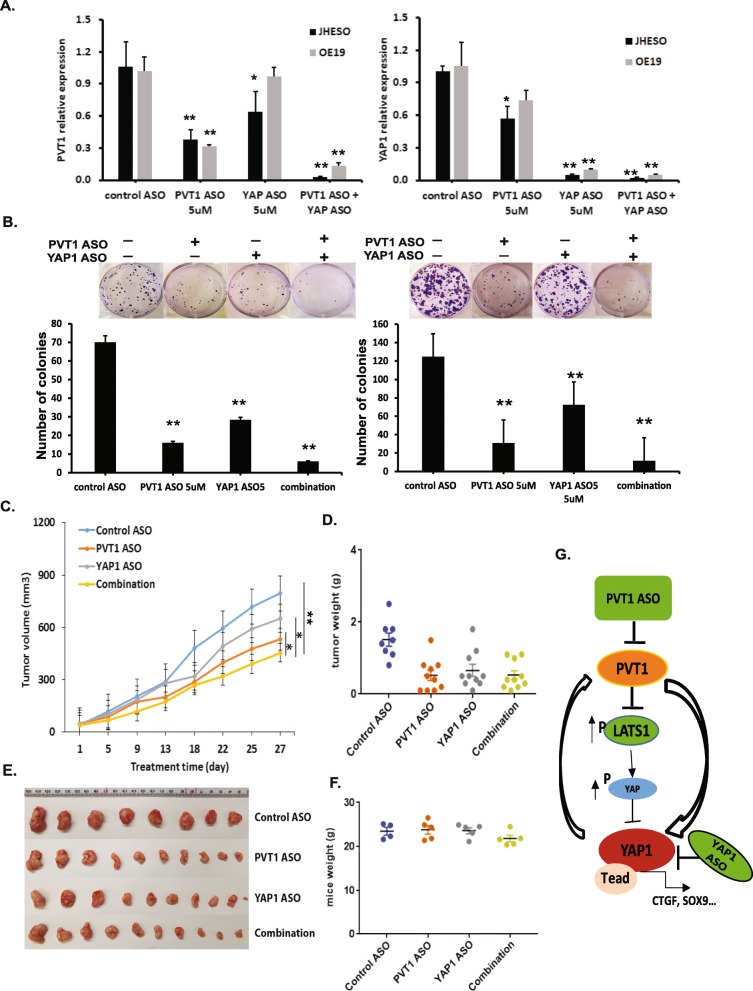
Simultaneous inhibition of PVT1 and YAP1 by their ASOs had better anti-tumor activity on EAC cells in vitro and in vivo. **a.** Expression of PVT1(left) and YAP1 (right) were detected in JHESO and OE19 cells treated with PVT1 ASOs, YAP1 ASOs or their combination by real-time q-PCR. **b.** Colony formation of JHESO (left) and OE19 (right) cells were significantly suppressed by PVT1 and YAP1 ASOs alone or in combination. **c**. Average tumor volume were demonstrated in mice that treated with PVT1 ASO, YAP1 ASO and their combination for 4 weeks. **d**. Tumors weights at the termination of the experiment were shown. **e** & **f.** Representative tumors (**e**) and mice weight (**f**) after 4 weeks were calculated as described as Materials&Methods. Error bars, mean ± SEM. E. *, *P* < 0.05; **, *P* < 0.01. **g.** Proposed model by which mutual regulation of PVT1 and YAP1

To study whether combined inhibition of PVT1 and YAP1 has stronger anti-tumor activity in EAC, we conducted in vitro and in vivo experiments using PVT1 and YAP1 specific ASOs. As shown in Fig. 6a, although either PVT1 ASO or YAP1 ASO alone can markedly suppress PVT and YAP1 expression, respectively, there was a simultaneous inhibition of the two targets with the combination both ASOs. Functionally, when JHESO and OE19 EAC cells were treated with PVT1 or YAP1 ASOs, colony formation demonstrated a substantial reduction in the number and average size of colonies in the ASO treatment group. Moreover, there were much fewer colonies in both JHESO and OE19 cells with the combination treatment compared to EAC cells treated with either ASO alone (Fig. 6b & Additional file 2: Figure S2B). These data suggested that blocking both PVT1 and YAP1 resulted in a synergistical inhibition of EAC cell growth in vitro.

To evaluate the antitumor effect in vivo, we tested anti-tumor effects of PVT1- and YAP1-specific ASOs alone or in combination of PVT1 and YAP1 specific ASOs in JHESO xenograft mice model. Nude mice were randomly assigned to one of four groups (treated with control ASO, PVT1 ASO, YAP1 ASO, and combination, respectively) after subcutaneous injection of JHESO cells. The in vivo xenograft experiments showed that simultaneous treatment using both ASOs markedly attenuated tumor growth compared to either ASO administration alone (Fig. 6c-e). There was no apparent change in body weight (Fig. 6f), which implied combination treatment is relatively nontoxic to these animals. In addition, qPCR results in these mouse tumors showed that expression of PVT1 and YAP1 in combination group were significantly reduced than either group alone (Additional file 3: Figure S3). Taken together, these results suggested that the mutual regulation of PVT1 and YAP1 exists in EAC cells and simultaneous inhibition of PVT1 and YAP1 lead to more effective suppression of EAC tumor growth than that of either gene alone. Thus, combination inhibition of YAP1 and PVT1 using their specific ASOs provide a novel therapeutic strategy for EAC (Fig. 6g).

## Discussion

PVT1 has been implicated in various cancer types [27]. However, its precise role in EAC remains unclear. In the present study, we verified that amplification of PVT1 gene is a common event in EAC. Moreover, PVT1 lncRNA is highly expressed in EAC tissues compared with normal esophageal tissues and BE. Higher expression of PVT1 lncRNA was closely correlated with higher tumor stage and poor prognosis. In vitro and in vivo assays demonstrated that inhibition of PVT1 achieved by PVT1 ASOs could inhibit EAC cell proliferation, invasion, reduced CSC-related characteristics, and delayed tumor growth. Furthermore, we demonstrated, for the first time, that there is mutual regulation of PVT1 and YAP1 in EAC cells and that co-suppression of PVT1 and YAP1 by their specific ASO led to more effective suppression of EAC tumor growth in vitro and in vivo.

Chromosome translocation was the first identified abnormality of PVT1 in tumors (Burkitt lymphoma) [28]. However, in many solid tumors, amplification and gain in copy number are the most frequent genetic alterations of PVT1 [29–31]. Notably, PVT1 co-amplification with MYC has been widely investigated considering they are located on the same chromosomal region (8q24) they are located on. Indeed, PVT1 lncRNA has been proven to play an oncogenic function by protecting MYC protein from phosphorylation-mediated degradation in breast cancer [7]. On the other hand, the promoter of PVT1 gene limits MYC transcription by competing in cis for the use of specific enhancers in a manner independent of PVT1 lncRNA [32]. Although both PVT1 and MYC coexist in 8q24, are amplified in many tumor types and are both highly upregulated in our EAC patient-cohort, we found that upregulation of PVT1 is highly associated with advanced stage and poor prognosis, while MYC expression was less relevant to poor survival. This further validated that PVT1 plays a more critical role than MYC in EAC progression. Furthermore, PVT1 lncRNA also exerts function through direct interaction with regulatory proteins (STAT3 [8], FOXM1 [10], KLF5 [31], NOP2 [33], etc.) and acts as competing endogenous RNAs (ceRNA) (miR-200 family [34, 35], miR-20a-5p [36]). These phenomena reflect that PVT1 has a diverse function relying on its complex structures and molecular interactions.

In the present study, we found the crosstalk between PVT1 lncRNA and the Hippo signaling pathway. A significant positive correlation between PVT1 and YAP1 expression was found in the patients with EAC. Mechanistically, PVT1 lncRNA knockdown increased phosphorylated LATS1 and phosphorylation of YAP1 which led to inactivated YAP1 leading to tumor growth inhibition. On the other hand, the PVT1 levels were significantly repressed in YAP1 knockout cells but enhanced in EAC cells with inducible YAP1 overexpression indicating a positive feedback regulation of PVT1 by YAP1. Unlike previous report by Xu M et al. that suggested FOXM1 regulation of PVT1 through direct binding to its promoter [10], we did not detect PVT1 promoter by the ChIP assay using YAP1 antibody, although there was a potential YAP1/TEAD binding motif (CATTCC) at PVT1 promoter upstream of 3.81 k from TSS of PVT1. However, the RNA-immunoprecipitation (RIP) assay showed that YAP1 was directly associated with PVT1. The direct interaction between YAP1 and PVT1 may lead to PVT1 stabilization and accumulation in EAC cells and therefore induce positive regulation. The detailed mechanism of the crosstalk between YAP1 and PVT1 remains unclear and warrants further investigation.

YAP1 is a downstream nuclear effector of the Hippo signaling pathway, which is involved in development, growth, and homeostasis. YAP1 protein has emerged as a promising therapeutic target in varies of cancer types. Our previous study demonstrated that YAP1 confers CSCs properties to nontumorigenic cells and cancer cells by regulating SOX9 in esophageal cancer [18]. Additionally, YAP1 upregulates EGFR expression and increases cell proliferation and therapy resistance in esophageal cancer [19]. In this study, we investigated the cooperative antitumor effect through knockdown of PVT1 and YAP1 using their specific ASOs. Our data showed that the ASOs, specific to PVT1 or YAP1, could achieve significant anti-tumor effect in vitro and in vivo. Furthermore, simultaneously blocking PVT1 and YAP1 is more effective than targeting either alone.

In summary, PVT1 lncRNA seems to play a critical role in EAC progression. In many solid cancers, PVT1 and YAP1 have been found to play an oncogenic role, and inhibition of either could be achieved by their specific ASOs and provide obvious antitumor activity. Most importantly, we demonstrated that there is a positive feedback regulation between PVT1 and YAP1. Thus, the combination of targeting PVT1 and YAP1 using their specific ASOs represents a promising therapeutic strategy for EAC.

## Additional files

Additional file 1: **Figure S1.** Analysis of the correlation between expression of MYC and clinical characteristics. Expression of MYC is not associated with stage, tumor grade, and tumor subtypes in EAC patients. (TIF 194 kb)

Additional file 2: **Figure S2.** Effects of additional PVT1 ASO (ASO6) in suppression of PVT1 level and colony formation. A. PVT1 ASO6 reduced PVT1 expression in dose-dependent manner in three EAC cell lines (OE19, FLO1, and JHESO). B. Colony formation of JHESO (left) and OE19 (right) cells was significantly suppressed by PVT1 and YAP1 ASOs alone or in combination. (TIF 1623 kb)

Additional file 3: **Figure S3.** The expression of PVT1 (top) and YAP1 (bottom) was measured by qPCR in PDX tumor tissues showed that significant reduction when treated with PVT1 and YAP1 ASOs alone or in combination. (TIF 118 kb)

Additional file 4: **Figure S4.** RIP assay in JSHEO and SKGT-4 cells using the YAP antibody to test the association between YAP and PVT1 by amplifying PVT1 using two individual PVT1 primers. **A**. PVT1 primer pair 1. B. PVT1 primer pair 2. (TIF 718 kb)

Additional file 5: **Table S1.** Primer sequences used for qPCR in the present study (DOCX 12 kb)

Additional file 6: **Table S2.** Information for antibodies used in western blot and immunofluorescent staining. (DOCX 12 kb)

## Data Availability

The datasets used and/or analyzed and materials used during the current study are available from the corresponding author on reasonable request.

## Abbreviations

ASOs: antisense oligonucleotides
ceRNA: competing endogenous RNA
CIN: chromosomal instability
CNVs: copy number variations
CSC: cancer stem cell
EAC: Esophageal adenocarcinoma
EC: Esophageal cancer
ESCA: Esophageal carcinoma
ESCC: esophageal squamous cell carcinoma
LncRNA: Long non-coding RNA
PVT1: plasmacytoma variant translocation 1
qPCR: quantitative real-time PCR
TCGA: The Cancer Genome Atlas
