# Correction to: LncRNA PVT1 up-regulation is a poor prognosticator and serves as a therapeutic target in esophageal adenocarcinoma

**DOI:** 10.1186/s12943-021-01351-5

**Published:** 2021-03-25

**Authors:** Yan Xu, Yuan Li, Jiankang Jin, Guangchun Han, Chengcao Sun, Melissa Pool Pizzi, Longfei Huo, Ailing Scott, Ying Wang, Lang Ma, Jeffrey H. Lee, Manoop S. Bhutani, Brian Weston, Christopher Vellano, Liuqing Yang, Chunru Lin, Youngsoo Kim, A. Robert MacLeod, Linghua Wang, Zhenning Wang, Shumei Song, Jaffer A. Ajani

**Affiliations:** 1 Departments of Gastrointestinal Medical Oncology, The University of Texas MD Anderson Cancer Center, 1515 Holcombe Blvd, Houston, TX 77030 USA; 2 Department of Surgical Oncology and General Surgery, First Hospital of China Medical University, Shenyang, 110001 People’s Republic of China; 3 Departments of Genomic Medicine, The University of Texas MD Anderson Cancer Center, Houston, TX 77030 USA; 4 Departments of Molecular & Cellular Oncology, The University of Texas MD Anderson Cancer Center, Houston, TX 77030 USA; 5 Departments of Gastroenterology&Hepatology, The University of Texas MD Anderson Cancer Center, Houston, TX 77030 USA; 6 Center for Co-Clinical Trial, The University of Texas MD Anderson Cancer Center, Houston, TX 77030 USA; 7 Ionis Pharmaceuticals, Inc., 2855 Gazelle Court, Carlsbad, CA 92010 USA

**Correction to: Mol Cancer (2019) 18:141**

**https://doi.org/10.1186/s12943-019-1064-5**

Following publication of the original article [1], the authors identified minor errors in image-typesetting in Fig. 3; specifically the left hand side of Fig. 3d.

**Fig. 3:**
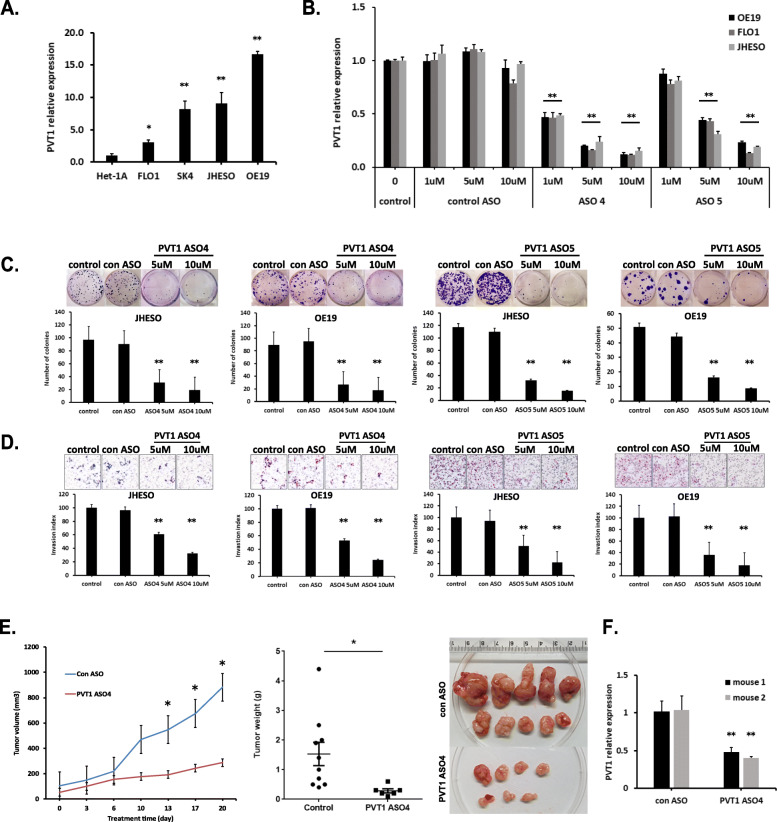
PVT1 suppression by PVT1 ASOs inhibits EAC cells growth in vitro and in vivo. **a** PVT1 expression in EAC cell lines (FLO1, SK-GT-4, JHESO, and OE19) and normal esophageal epithelial cell line HET-1A were determined by realtime qPCR analysis. **b** PVT1 expression was analyzed by qPCR in PVT1 ASOs treated EAC cell lines. PVT1 specific antisense oligonucleotides (ASO4 and ASO5) reduced PVT1 expression in dose-dependent manner in three EAC cell lines. c and d. Inhibition of PVT1 by ASOs significantly suppressed colony formation (**c**) and decreased cell invasion (**d**) in both JHESO and OE19 cells in two individual PVT1 ASOs respectively. **e** Average tumor volume in mice that treated with PVT1 ASO4 or control ASO via subcutaneous injection for 3 weeks (left). Actual tumor weights were retrieved from the mice at the termination of the experiment (middle). Representative tumors after scarified were shown (right). **f** The expression of PVT1 in mouse tumor tissues measured by qPCR has been shown significant reduction upon treatment with PVT1 ASO4 compared to the control group. Error bars, mean ± SEM. *, *P* < 0.05; **, *P* < 0.01

The corrected figure is given below. The correction does not have any effect on the results or conclusions of the paper.
