# Supplementary figures and images for: LncRNA PVT1 up-regulation is a poor prognosticator and serves as a therapeutic target in esophageal adenocarcinoma
Source: Mol Cancer. 2019 Oct 10;18:141. doi: 10.1186/s12943-019-1064-5 (PMC6785865; doi:10.1186/s12943-019-1064-5)

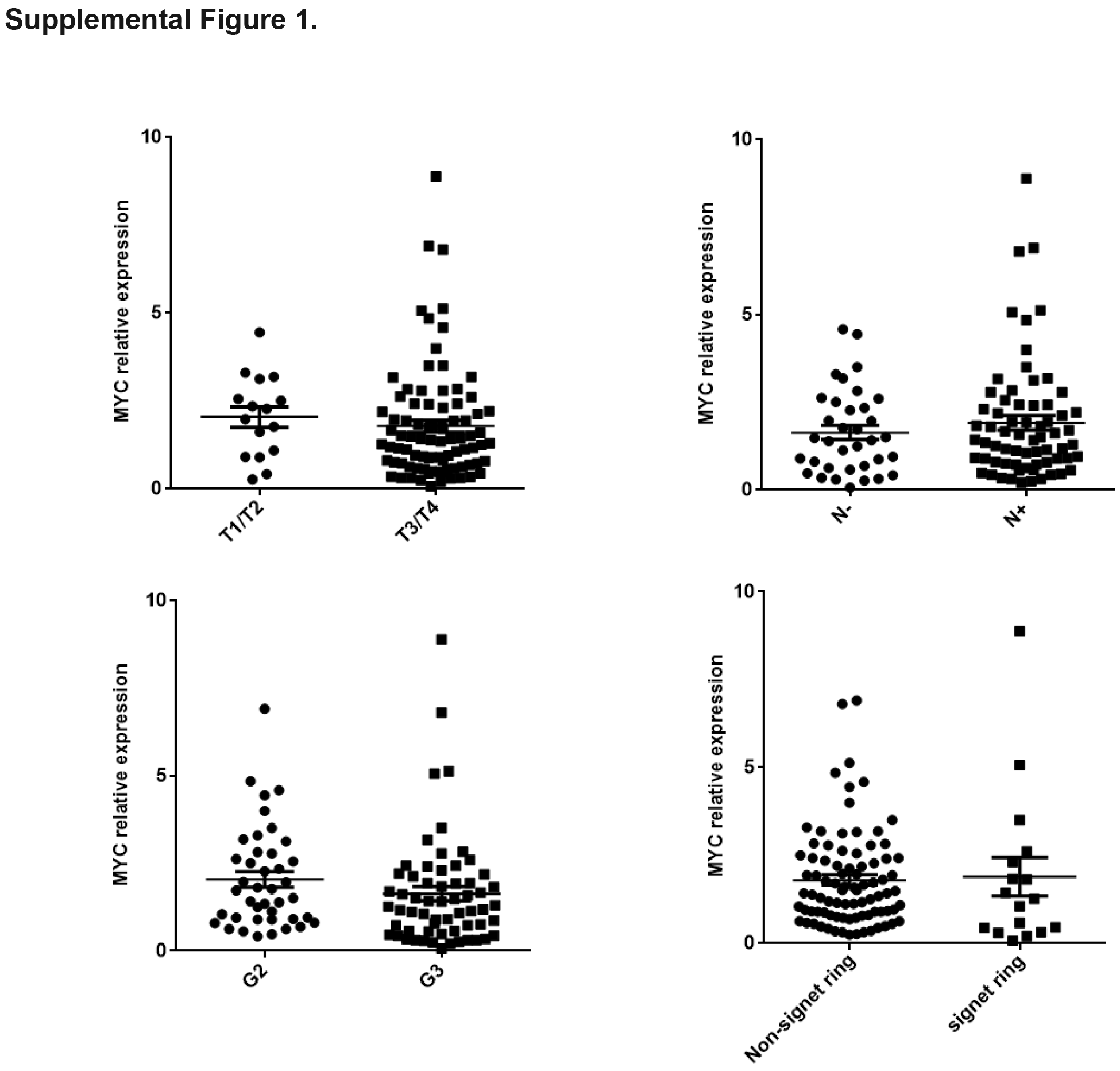

Supplement: Supplementary file 1 — Figure S1. Analysis of the correlation between expression of MYC and clinical characteristics. Expression of MYC is not associated with stage, tumor grade, and tumor subtypes in EAC patients. (TIF 194 kb) [file 12943_2019_1064_MOESM1_ESM.tif]

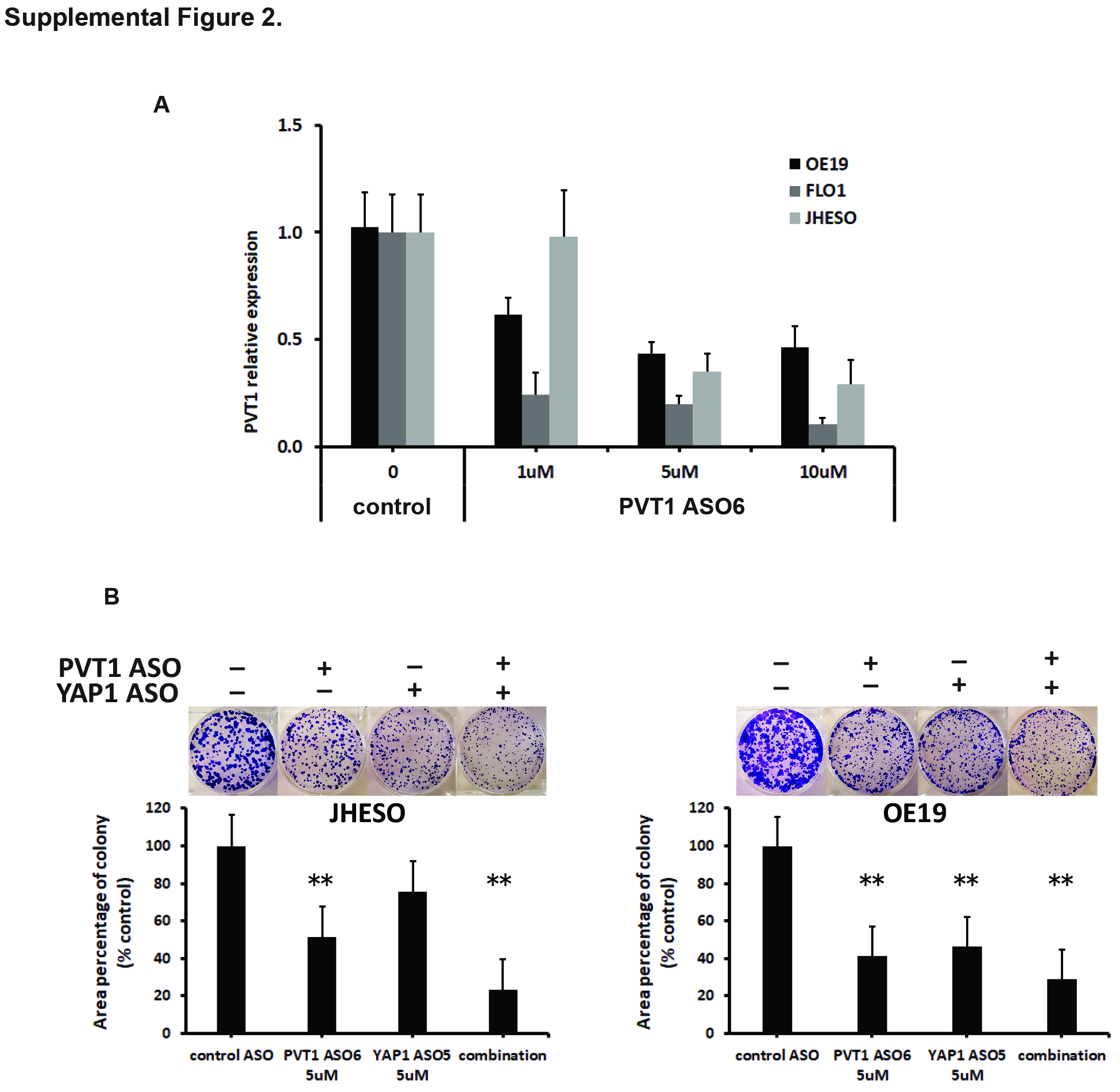

Supplement: Supplementary file 2 — Figure S2. Effects of additional PVT1 ASO (ASO6) in suppression of PVT1 level and colony formation. A. PVT1 ASO6 reduced PVT1 expression in dose-dependent manner in three EAC cell lines (OE19, FLO1, and JHESO). B. Colony formation of JHESO (left) and OE19 (right) cells was significantly suppressed by PVT1 and YAP1 ASOs alone or in combination. (TIF 1623 kb) [file 12943_2019_1064_MOESM2_ESM.tif]

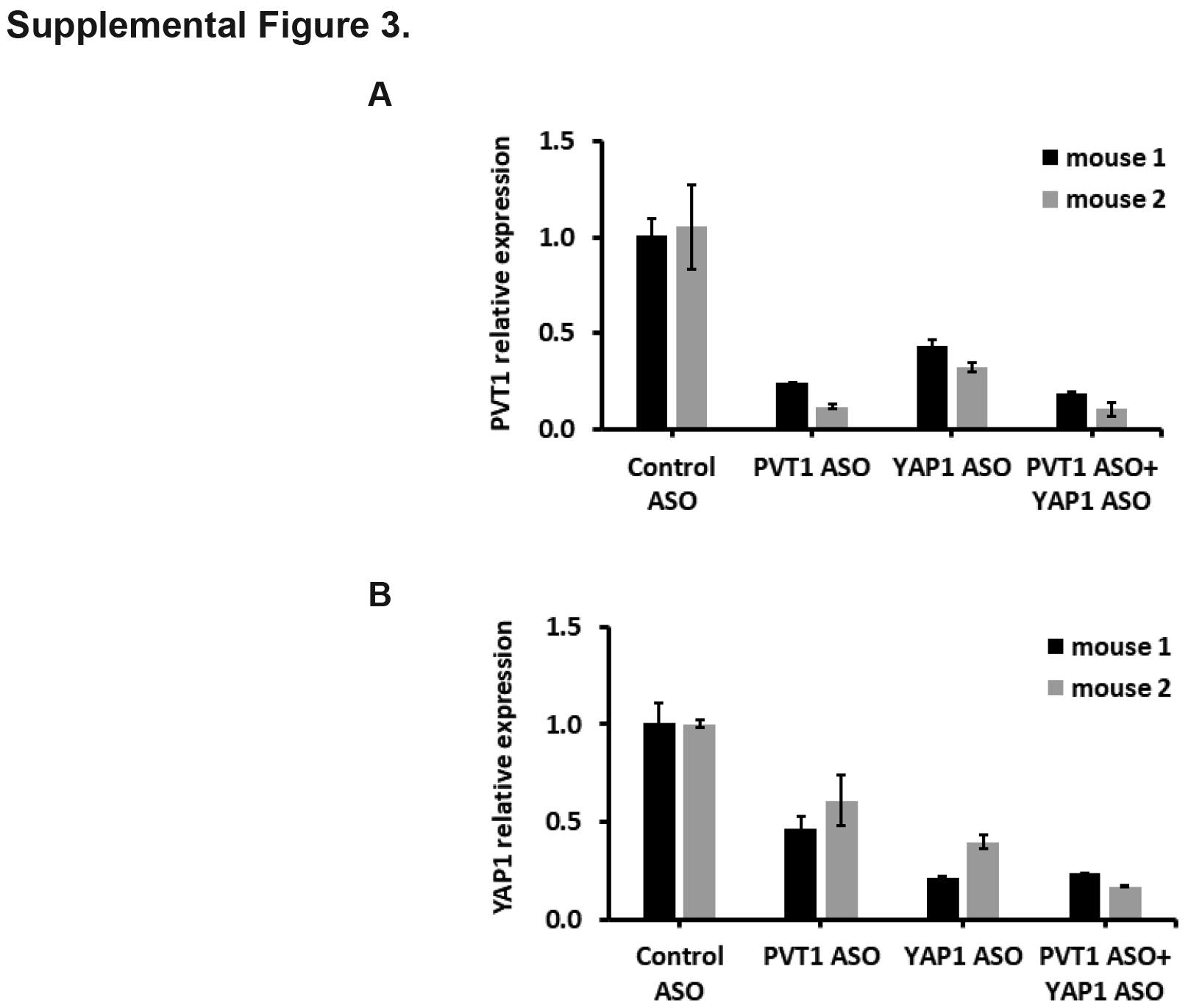

Supplement: Supplementary file 3 — Figure S3. The expression of PVT1 (top) and YAP1 (bottom) was measured by qPCR in PDX tumor tissues showed that significant reduction when treated with PVT1 and YAP1 ASOs alone or in combination. (TIF 118 kb) [file 12943_2019_1064_MOESM3_ESM.tif]

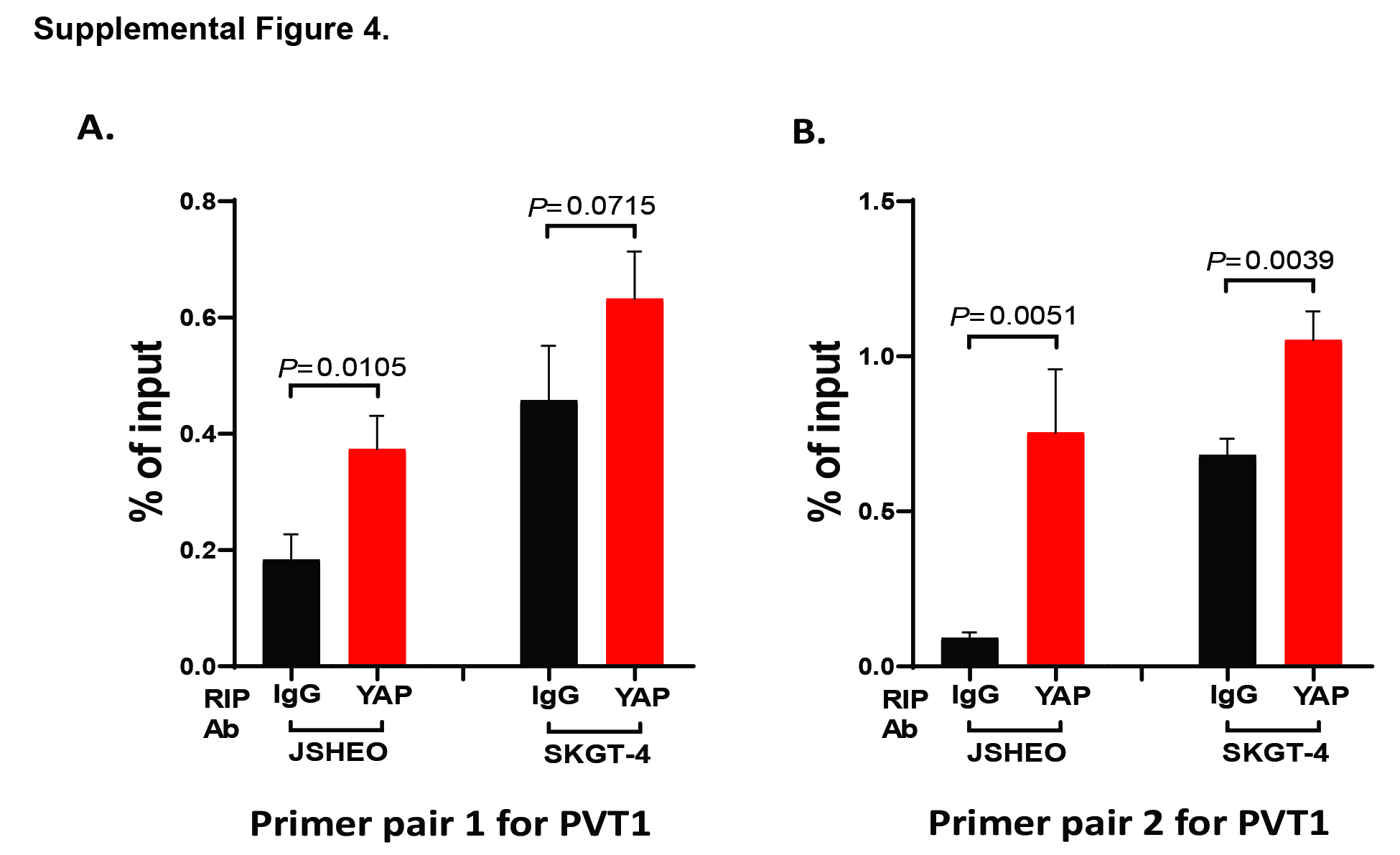

Supplement: Supplementary file 4 — Figure S4. RIP assay in JSHEO and SKGT-4 cells using the YAP antibody to test the association between YAP and PVT1 by amplifying PVT1 using two individual PVT1 primers. A. PVT1 primer pair 1. B. PVT1 primer pair 2. (TIF 718 kb) [file 12943_2019_1064_MOESM4_ESM.tif]
