# Supplementary material for: LncRNA PVT1 up-regulation is a poor prognosticator and serves as a therapeutic target in esophageal adenocarcinoma
Source: Mol Cancer. 2019 Oct 10;18:141. doi: 10.1186/s12943-019-1064-5 (PMC6785865; doi:10.1186/s12943-019-1064-5)
Supplement: Supplementary file 5 — Table S1. Primer sequences used for qPCR in the present study (DOCX 12 kb) [file 12943_2019_1064_MOESM5_ESM.docx]

**Supplemental table 1.**

**Primer sequences used for qPCR in the present study**

1. PVT1:

Fw, 5’-TGAGAACTGTCCTTACGTGACC -3’;

Rev, 5’ -AGAGCACCAAGACTGGCTCT -3’,

2. YAP1:

Fw, 5’- CTGTCCCAGATGAACGTCAC -3’;

Rev, 5’ - TTCTCTGGTTCATGGCAAAA -3’.

3. MYC:

Fw, 5’- CTCCTCCTCGTCGCAGTAGA -3’;

Rev, 5’ - GCTGCTTAGACGCTGGATTT -3’.

4. SOX9:

Fw, 5’- GTAATCCGGGTGGTCCTTCT -3’;

Rev, 5’ - GTACCCGCACTTGCACAAC -3’.

5. CTGF:

Fw, 5’- GTGTGCACCGCCAAAGATGG -3’;

Rev, 5’ - AGGCACGTGCACTGGTACTT -3’.

6. GAPDH:

Fw, 5’-TCTAGACGGCAGGTCAGGTC -3’;

Rev, 5’ -ACCCAGAAGACTGTGGATGG -3’.
