# Supplementary material for: LncRNA PVT1 up-regulation is a poor prognosticator and serves as a therapeutic target in esophageal adenocarcinoma
Source: Mol Cancer. 2019 Oct 10;18:141. doi: 10.1186/s12943-019-1064-5 (PMC6785865; doi:10.1186/s12943-019-1064-5)
Supplement: Supplementary file 6 — Table S2. Information for antibodies used in western blot and immunofluorescent staining. (DOCX 12 kb) [file 12943_2019_1064_MOESM6_ESM.docx]

**Supplemental table 2.**

**Information for antibodies used in western blot and immunofluorescent staining**

| Name | Vender | Cat No. | Species | Dilution |
| --- | --- | --- | --- | --- |
| YAP1 | Cell signaling | 4912 | Rabbit polyclonal IgG | 1:1000 |
| Phosphor YAP1 ser127 | Cell signaling | 3477 | Rabbit polyclonal IgG | 1:1000 |
| Phosphor LATS1 ser909 | Cell signaling | 9157 | Rabbit polyclonal IgG | 1:1000 |
| β-actin | Abcam | Ab8226 | Mouse polyclonal IgG | 1:5000 |
